# An Economic Analysis of Strategies to Control *Clostridium Difficile* Transmission and Infection Using an Agent-Based Simulation Model

**DOI:** 10.1371/journal.pone.0152248

**Published:** 2016-03-31

**Authors:** Richard E. Nelson, Makoto Jones, Molly Leecaster, Matthew H. Samore, William Ray, Angela Huttner, Benedikt Huttner, Karim Khader, Vanessa W. Stevens, Dale Gerding, Marin L. Schweizer, Michael A. Rubin

**Affiliations:** 1 Veterans Affairs Salt Lake City Health Care System, Salt Lake City, UT, United States of America; 2 Department of Internal Medicine, University of Utah School of Medicine, Salt Lake City, UT, United States of America; 3 Infection Control Program, Geneva Hospitals, Geneva, Switzerland; 4 Department of Pharmacotherapy, University of Utah College of Pharmacy, Salt Lake City, UT, United States of America; 5 Edward Hines Jr. VA Hospital, Hines, IL, United States of America; 6 Iowa City Veterans Affairs Health Care System, Iowa City, IA, United States of America; 7 Department of Internal Medicine, University of Iowa Carver College of Medicine, Iowa City, IA, United States of America; Cleveland Clinic, UNITED STATES

## Abstract

**Background:**

A number of strategies exist to reduce *Clostridium difficile* (*C*. *difficile*) transmission. We conducted an economic evaluation of “bundling” these strategies together.

**Methods:**

We constructed an agent-based computer simulation of nosocomial *C*. *difficile* transmission and infection in a hospital setting. This model included the following components: interactions between patients and health care workers; room contamination via *C*. *difficile* shedding; *C*. *difficile* hand carriage and removal via hand hygiene; patient acquisition of *C*. *difficile* via contact with contaminated rooms or health care workers; and patient antimicrobial use. Six interventions were introduced alone and "bundled" together: (a) aggressive *C*. *difficile* testing; (b) empiric isolation and treatment of symptomatic patients; (c) improved adherence to hand hygiene and (d) contact precautions; (e) improved use of soap and water for hand hygiene; and (f) improved environmental cleaning. Our analysis compared these interventions using values representing 3 different scenarios: (1) base-case (BASE) values that reflect typical hospital practice, (2) intervention (INT) values that represent implementation of hospital-wide efforts to reduce *C*. *diff* transmission, and (3) optimal (OPT) values representing the highest expected results from strong adherence to the interventions. Cost parameters for each intervention were obtained from published literature. We performed our analyses assuming low, normal, and high *C*. *difficile* importation prevalence and transmissibility of *C*. *difficile*.

**Results:**

INT levels of the “bundled” intervention were cost-effective at a willingness-to-pay threshold of $100,000/quality-adjusted life-year in all importation prevalence and transmissibility scenarios. OPT levels of intervention were cost-effective for normal and high importation prevalence and transmissibility scenarios. When analyzed separately, hand hygiene compliance, environmental decontamination, and empiric isolation and treatment were the interventions that had the greatest impact on both cost and effectiveness.

**Conclusions:**

A combination of available interventions to prevent CDI is likely to be cost-effective but the cost-effectiveness varies for different levels of intensity of the interventions depending on epidemiological conditions such as *C*. *difficile* importation prevalence and transmissibility.

## Introduction

*Clostridium difficile* (*C*. *difficile*) infection (CDI) is one of the most important types of healthcare-associated infections worldwide. CDI can lead to diarrhea, fulminant colitis and death[1] and has been shown to significantly increase length of stay and hospital costs.[2] Since 2000, CDI incidence has been steadily increasing in the United States,[3,4] Canada,[5] and Europe.[6] In fact, recent data suggests that *C*. *difficile* has replaced methicillin-resistant *Staphylococcus aureus* as the most common cause of healthcare-associated infection.[7]

*C*. *difficile* is a gram-positive anaerobic bacillus that is acquired by the host through ingestion of spores.[8] These spores are often transmitted between patients by healthcare workers and the environment[9,10] and proliferate in the small intestine often due to disruption of normal gut flora following exposure to antibiotics.[8] While there is an urgent need for improved hospital infection control practice, the infection control community is highly divided on the most appropriate strategy to control transmission of *C*. *difficile* within healthcare institutions.[11]

Economic analyses play an important role in assisting decision makers in evaluating competing strategies and can provide insight into real-world feasibility. However, few economic analyses of *C*. *difficile*-related treatment or prevention strategies exist. Those that do, focus on a hypothetical vaccine,[12] treatment with antimicrobial agents such as vancomycin or fidaxomycin,[13,14] or admission screening for *C*. *difficile* colonization.[15]

Most cost-effectiveness analyses assume that the probability of exposure to the disease of interest is not affected by an intervention to treat or prevent it. These models are referred to as “static” because this assumption of independence between exposure to disease and the intervention causes disease exposure to remain unchanged over time. This assumption, while realistic for most diseases, is not valid for diseases due to transmissible organisms such as *C*. *difficile*.[16] Dynamic models, on the other hand, allow for interventions to decrease the risk of treated patients developing illness as well as to decrease the probability of exposure for other patients, thereby, more accurately representing the behavior of infectious diseases.[17]

The objective of this study was to perform a dynamic cost-effectiveness analysis using a previously developed agent-based simulation model (ABM) to compare the costs and outcomes of 6 current and novel CDI control strategies for the hospital setting. The simulation was designed to explore each intervention strategy alone and in various combinations, representing different hypothetical intervention "bundles."

## Methods

### Agent-based simulation

ABMs are a class of computer modeling that is useful for studying complex dynamic systems such as healthcare delivery environments.[18] ABMs allow for a realistic representation of these systems and provide inexpensive laboratories where decision makers can conduct “what-if” experimentation. We constructed our ABM are in Anylogic 6.5 (XJ Technologies, St. Petersburg, Russia) using Java-based graphical editing tools to create replicated agents and their associated parameters, variables, and state-transition diagrams or statecharts.

### Model

Agent classes included patients, nurses, physicians, and rooms. Each agent existed in explicit “states” governed by statecharts; transitions between states were treated as discrete events that occur probabilistically. The various components of the model were divided into a series of static and dynamic "sub-models”. Hospital room occupancy was governed by a submodel directing patient flow regarding admission, room transfer, and discharge. Patients moved within and across wards in the hospital. Rooms held dynamic quantities of *C*. *difficile*, increasing due to shedding and depletion over time. Patients could acquire *C*. *difficile* asymptomatically through contact with healthcare workers or the room environment, and could then progress to symptomatic CDI with shedding of organisms into the room environment. Antibiotics were given to patients during their stay and impacted the acquisition, progression to symptomatic CDI, and organism shedding. Treatment also led to decreased shedding into the environment. Each iteration of the model simulated approximately 20,000 patient admissions. The model did not include complications of CDI such as colectomy or death. In addition, recurrent CDI was not considered in the model and treatment was assumed to always be curative. More details on the model can be found in Rubin et al (2013)[19] and in the S1 Appendix.

### Intervention strategies

We used our ABM to study the impact of 6 interventions and policies for reducing *C*. *difficile* transmission and infection: (1) aggressive and early testing for *C*. *difficile* (TIME), (2) empiric isolation and treatment for suspected cases of CDI (ISOL), (3) improved adherence with hand hygiene (HAND), (4) improved adherence with barrier precautions for contacts with CDI patients (BARR), (5) improved use of soap and water for hand hygiene after contacts with CDI patients (SOAP), and (6) improved environmental decontamination (DCON).

The economic analysis compared the costs and the outcomes associated with 3 implementation levels of each of the bundled infection control strategies: (I) a base-case (BASE) level, reflecting current realities in a typical hospital not employing interventions specifically targeting *C*. *difficile*, (II) an intervention (INT) level, representing an improvement over BASE parameter values that could reasonably be expected from typical adherence to an effort focusing on that particular strategy, and (III) an optimal (OPT) level, which represents maximum effects that can be reasonably expected from strong adherence to an intensive and aggressive intervention campaign.

Our primary economic analysis consisted of comparing the costs and effectiveness of the 6 interventions where each of the interventions was set to either BASE, INT, and OPT levels. In a secondary analysis, we examined the economic consequences of different combinations of the 3 levels for each of the 6 interventions.

### Input parameters

#### Interventions

For each of the 6 interventions, we identified key parameters to represent BASE, INT, and OPT levels. The values of these parameters are presented in Table 1. The HAND intervention consisted of increases in adherence for both nurses and physicians in both isolation and non-isolation rooms and both before and after patient contact for INT and OPT relative to BASE.[20–23]

**Table 1:** Key parameter inputs to simulation model.

	Strategy									
	BASE			INT			OPT			
Parameter	Mean	LL	UL	Mean	LL	UL	Mean	LL	UL	Source
**Interventions**										
*Hand hygiene*										
Non-isolation rooms										
Nurses										
Before patient contact	30	20	40	60	53	67	80	75	85	[20–23]
After patient contact	50	40	60	74	67	81	90	85	95	[20–23]
Physicians										
Before patient contact	20	10	30	50	43	57	70	65	75	[20–23]
After patient contact	40	30	50	64	57	71	80	75	85	[20–23]
Isolation rooms										
Nurses										
Before patient contact	50	40	60	74	67	81	90	85	95	[20–23]
After patient contact	70	60	80	82	75	89	90	85	95	[20–23]
Physicians										
Before patient contact	30	20	40	54	47	61	70	65	75	[20–23]
After patient contact	50	40	60	68	61	75	80	75	85	[20–23]
*Soap and water*	60			80			90			DA, EX
*Contact precautions*	60			75			90			[24]
*Environmental cleaning*										
Routine daily (e.g., detergent-based)	27.5	24	28	30	29	32	35	33	38	[25–29]
Routine terminal (e.g., detergent-based)	35	33	37	40	38	41	42.5	42	44	[25–29]
Deep terminal (e.g., chlorine-based)	70	66	74	80	76	84	90	86	94	[25–29]
*Testing*	1.5	1.25	1.75	1	1.2	0.8	0.5	0.75	0.25	[30–32]
*Empiric isolation and treatment*	No			Yes			Yes			Model
**Epidemiologic conditions**										
*Importation*	2			7.5			15			[33] EX
*Transmissibility*				*see Methods *for details*						DA, EX

*Note*: For hand hygiene intervention, values = % adherence in non-isolation/isolation rooms. For soap and water intervention, values = % use of each. For contact precautions intervention, values = % adherence. For environmental cleaning intervention, values = % organism reduction. For testing intervention, values = mean time (days) from symptom to test order. For importation, values = % prevalence.

DA = analysis of local and national data, EX = subject matter expert opinion.

BASE = level of parameters in a typical hospital not employing interventions specifically targeting *C*. *difficile*.

INT = intervention level of parameter.

OPT = optimal level of parameter.

For SOAP, we assumed that an intervention would increase the probability that, after coming in contact with a patient known to have CDI, a healthcare worker used soap and water rather than an alcohol-based hand rub. To operationalize this in the model, we used local and national Department of Veterans Affairs (VA) data to obtain estimates for the percentage of soap and water used in VA hospitals.

When patients with suspected CDI were isolated in the model, compliance with barrier precautions were implemented probabilistically. We set the BASE level of adherence as well as the increases in this level for INT and OPT for contact precautions for isolation rooms based on a published study for the BARR intervention.[24]

In our model, rooms were cleaned routinely on a daily basis and a terminal cleaning was performed after each discharge. This cleaning was done using quaternary ammonium compounds, which have limited effect on *C*. *difficile*, for rooms with non-CDI patients. When patients had CDI, terminal cleaning was performed using hypochlorite. For the DCON intervention, we assumed increased efficacy due to improvements in cleaning effort and solution exposure.[25–29]

Finally, for INT and OPT levels, the mean time from symptoms to test order was reduced for the TIME intervention[30–32] and patients with suspected *C*. *difficile* infection were isolated and treated in the ISOL intervention.

#### Epidemiologic conditions

The effectiveness of infection control interventions can depend heavily on the underlying epidemiologic conditions within a certain environment. Our model included parameters that characterized the importation and transmissibility of *C*. *difficile*, both of which could take on 3 levels: low, medium, and high. The importation parameter values[33] are shown in Table 1.

The transmissibility parameter in the model was a function of the probability of transmission during a contact between a healthcare worker and a patient. This parameter value was tuned internally in the model to produce *C*. *difficile* acquisition and infection rates to correspond to each of the 3 transmissibility levels. For additional model parameters, please see Rubin et al (2013).[19]

#### Costs and quality of life

Table 2 shows the values of cost and quality of life inputs. Costs were adjusted to 2011 US dollars and taken from the hospital perspective. The cost inputs for the hand hygiene intervention included the variable cost of alcohol-based hand sanitizer ($0.07 per application)[34] and the fixed costs of a campaign to promote improved hand hygiene ($54,284),[35] which included salaries of personnel and office supplies related to the promotion activities. The cost of improved barrier precautions included the costs associated with gloves ($0.09 per pair) and gowns ($0.92 each).[36] The cost of environmental decontamination, which included both costs of the cleaning agents and personnel time, consisted of routine daily cleaning ($22.33), routine terminal cleaning ($36.52), and deep terminal cleaning ($172.95).[37] Patients were tested for the presence of *C*. *difficile* using a polymerase chain reaction (PCR) test ($7.66 per test), which required time from a laboratory technician.[38] Finally, symptomatic patients were treated with a course of oral metronidazole ($57) or vancomycin ($1,347)[15] and a *C*. *difficile* infection was assumed to cost $11,056.[39]

**Table 2:** Cost and utility parameters.

Parameter	Cost	LL	UL	Source
*Hand hygiene*				
Variable cost per application	$0.07	$0.03	$0.13	[34]
Fixed costs for hand hygiene promotion campaign				
BASE	$0	$0	$0	[35]
INT	$27,142	$26,598	$27,692	[35]
OPT	$54,284	$53,451	$55,123	[35]
*Soap and water*				
Variable cost per application	$0.07	$0.03	$0.13	[34]
*Contact precautions*				
Gloves (per pair)	$0.09	$0.04	$0.16	[36]
Gown	$0.92	$0.42	$1.60	[36]
*Environmental cleaning*				
Routine daily cleaning				
BASE	$17.55	$6.87	$33.16	[37]
INT	$19.33	$7.60	$36.43	[37]
OPT	$22.33	$8.73	$42.20	[37]
Routine terminal cleaning				
BASE	$30.08	$11.77	$56.84	[37]
INT	$34.38	$13.44	$64.98	[37]
OPT	$36.52	$14.28	$69.03	[37]
Deep terminal cleaning				
BASE	$134.52	$52.60	$254.23	[37]
INT	$153.74	$60.12	$290.55	[37]
OPT	$172.95	$67.63	$326.85	[37]
*Testing*				
PCR test	$7.66	$2.99	$14.48	[38]
Technician wage	$17.96	$7.03	$33.93	BLS
Technician time (minutes)	11.0	4.2	21.0	[38]
*Treatment of symptomatic patients*				
Vancomycin	$1,347	$527	$2,545	[15]
Metronidozole	$57	$22	$109	[15]
*Healthcare-associated Clostridium difficile infection*	$11,056	$8,933	$13,299	[39]
*Utility values*				
Age 45–54	0.87	0.80	0.90	[45]
Age 55–64	0.81	0.75	0.85	[45]
Age 65–74	0.77	0.72	0.82	[45]
Age 75–79	0.70	0.65	0.75	[45]
*Attributable mortality due to healthcare-associated Clostridium difficile infection*	0.051	0.038	0.068	[43,44]

*Note*: BLS = Bureau of Labor Statistics.

BASE = level of parameters in a typical hospital not employing interventions specifically targeting *C*. *difficile*.

INT = intervention level of parameter.

OPT = optimal level of parameter.

Effectiveness measures included CDIs averted and quality-adjusted life-years (QALYs), a commonly used metric in cost-effectiveness analyses that combines both duration and quality of life using utility weights that vary between 0 and 1. To our knowledge, no published values of the utility associated with CDI exist. Therefore, to construct QALYs, we followed other published studies[15] by using the utility of non-infectious diarrhea as a proxy measure of the utility of *C*. *difficile*-related diarrhea.[40–42] We assumed that the attributable mortality due to healthcare-associated CDI was 5.2%, which was calculated based on an overall mortality rate of 2% for hospitalized patients[43] and an odds ratio (95% confidence interval) of 2.62 (1.91–3.59) for death due to CDI based on a pooled analysis of published studies.[44] We adopted a lifetime horizon with a life expectancy of 78.7 years to incorporate differences in mortality stemming from differences in CDI rates for the different intervention strategies. QALYs occurring in future time periods were discounted at a rate of 3%. Assuming patients entered the model at 45 years of age and using age-based utility values for remaining years of life,[45] patients who did not die due to CDI were assumed to gain 18.0 discounted QALYs. Because all costs associated with the initial hospital stay were assumed to occur in the first year, we did not discount costs.

#### Analysis

To explore the cost-effectiveness of strategies in which all 6 interventions were set to the same level as a “bundle”, our primary analysis, we ran our model under the 3 different levels of importation and transmissibility. We ran a total of 100 iterations of the model for each strategy level (BASE, INT, and OPT), for each transmission parameter value (low, medium, and high), and for each importation parameter value (low, medium, and high) for a total of 2700 iterations.

For the secondary analysis, we allowed each of the 6 interventions to be set at different levels of BASE, INT, or OPT, leading to 486 different combinations (3 levels for HAND, SOAP, BARR, DCON, and TIME, and 2 levels for ISOL). We ran a total of 60 iterations of the model for each of these 486 different combinations for a total of 29,160 iterations.

The results from our model runs were presented as incremental cost-effectiveness ratios (ICERs), defined as the difference in the cost between two interventions divided by the difference in effectiveness of the same two interventions. The cost and effectiveness values used in the construction of the ICERs were the mean values across all relevant iterations.

Finally, we conducted probabilistic sensitivity analyses (PSAs) in which all parameter values were allowed to vary simultaneously through 2^nd^ order Monte Carlo simulations. The parameters that were varied in these PSAs included the probabilities associated with the HAND intervention as well as cost and utility parameters. In these analyses, 195 iterations were run for each strategy level, for each transmission parameter value, and for each importation parameter value for a total of 5265 iterations. In each iteration, the values for these parameters were drawn from distributions which were specified based on the characteristics specific to the parameter.[46] For instance, because probability and utility values can only take values between 0 and 1, we assigned a beta distribution. Healthcare costs, on the other hand, are constrained to be non-negative and are often heavily skewed due to a small number of extreme outliers. For this reason, we chose to represent cost parameters with a gamma distribution. The beta distribution is composed of an alpha and a beta parameter while the gamma distribution is composed of a scale and a shape parameter. The two parameters for both distributions are each composed of the mean and standard deviation statistics. The mean values and the 95% confidence intervals, from which the standard deviations were derived, are presented in Table 1 for each probability parameter used in the PSA and in Table 2 for each cost and utility parameter used in the PSA.

## Results

Model results for the primary analysis in the form of per-patient costs, QALYs, and CDIs per 10,000 patient-days are presented in Table 3. As intervention levels increased (i.e., from BASE to INT to OPT), rates of CDI decreased, suggesting that the bundled interventions were successful at containing and preventing the spread of *C*. *difficile* in the hospital. Per-patient costs ranged from $154 for BASE and low importation and transmission to $284 with BASE and high importation and transmission.

**Table 3:** Mean cost, QALYs, and infections per 10,000 patient days by BASE, INT, and OPT.

	Low Transmission			Medium Transmission			High Transmission		
Importation	Cost	Infections^a^	QALYs^b^	Cost	Infections^a^	QALYs^b^	Cost	Infections^a^	QALYs^b^
*Low importation*									
BASE	$154	1.8	18.0129	$165	3.4	18.0121	$198	8.1	18.0098
INT	$176	1.3	18.0132	$181	1.8	18.0129	$185	2.4	18.0126
OPT	$202	1.2	18.0132	$205	1.6	18.0130	$209	2.0	18.0128
*Medium importation*									
BASE	$171	3.8	18.0119	$196	7.5	18.0101	$242	14.2	18.0068
INT	$194	2.9	18.0124	$203	4.1	18.0118	$216	5.7	18.0110
OPT	$220	2.6	18.0125	$227	3.4	18.0121	$235	4.5	18.0116
*High importation*									
BASE	$203	7.8	18.0099	$243	3.6	18.0071	$284	19.6	18.0041
INT	$226	5.6	18.0111	$245	7.9	18.0099	$265	10.6	18.0086
OPT	$253	5.3	18.0112	$266	6.9	18.0104	$280	8.7	18.0095

Note: results depict the mean, per-patient values across 100 iterations with approximately 20,000 patient admissions per strategy level.

^*a*^*Clostridium difficile* infections per 10,000 patient-days.

^*b*^QALY = quality-adjusted life-years.

BASE = level of parameters in a typical hospital not employing interventions specifically targeting *C*. *difficile*.

INT = intervention level of parameter.

OPT = optimal level of parameter.

Table 4 presents the ICERs for the primary analysis. Compared to BASE level values for each bundle component, INT resulted in dominance (improved effectiveness and lower costs) for high transmission regardless of the importation level. The ICER for INT ranged from $616 to $80,118 per QALY for low and medium transmission levels. OPT had an ICER ranging from $15,628 to $923,269 per QALY across the levels of the epidemiological parameters.

**Table 4:** Results from cost-effectiveness analysis.

	Effectiveness measure = infections averted			Effectiveness measure = QALYs*^a^*		
		Transmission			Transmission	
* *Importation	Low	Medium	High	Low	Medium	High
*Low importation*						
BASE	-	-	-	-	-	-
INT	$36,936	$22,114	Dominant	$80,118	$19,892	Dominant
OPT	$434,024	$388,071	$112,865	$923,269	$189,776	$110,952
*Medium importation*						
BASE	-	-	-	-	-	-
INT	$10,980	$3,115	Dominant	$51,611	$4,272	Dominant
OPT	$95,788	$78,655	$26,176	$211,511	$73,780	$29,473
*High importation*						
BASE	-	-	-	-	-	-
INT	$6,963	$506	Dominant	$20,389	$616	Dominant
OPT	$56,243	$38,835	$13,978	$197,459	$41,531	$15,628

Note: this table shows incremental cost-effectiveness ratios for different levels of transmission and importation. A dominant strategy is both more effective and less costly than a comparator.

^*a*^QALY = quality-adjusted life-years.

BASE = level of parameters in a typical hospital not employing interventions specifically targeting *C*. *difficile*.

INT = intervention level of parameter.

OPT = optimal level of parameter.

**Fig 1:**
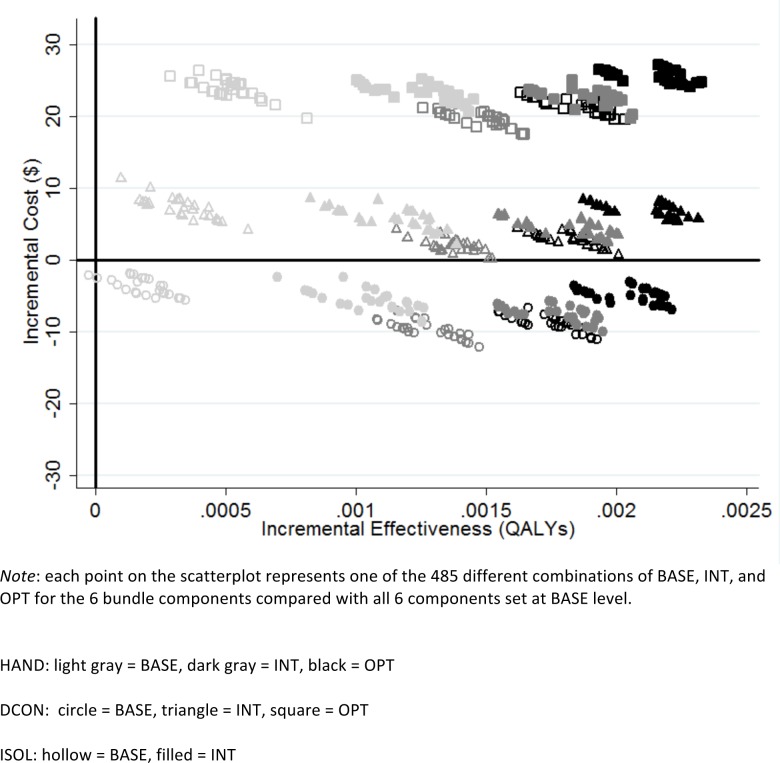
Scatterplot of incremental cost and effectiveness (*C*. *difficile* infections averted) of intervention strategies compared with BASE levels of all 6 bundle components. HAND: light gray = BASE, dark gray = INT, black = OPT; DCON: circle = BASE, triangle = INT, square = OPT; ISOL: hollow = BASE, filled = INT.

Fig 1 depicts the results from the analyses in which the level of each intervention varied independently (the secondary analysis). The resultant 486 combinations (3 levels for each component except ISOL which had 2 levels) allowed for 485 comparisons with all 6 interventions set at BASE as the comparison intervention. Each point on this scatterplot represents 1 of the 485 comparisons on the cost-effectiveness plane. The y-axis of this plane represents the difference in costs between two interventions and the x-axis represents the difference in effectiveness between two interventions. The points clustered based on the levels of 3 of the 6 bundle components: HAND, DCON, and ISOL. Therefore, for ease of presentation, only the levels of these components are depicted visually in the graph (by color, shape, and fill, respectively). This figure demonstrates that bundle combinations become more expensive but not much more effective (i.e., move to the north) as the DCON level increases. As HAND levels increase, bundle combinations become more effective without much impact on cost (i.e., move to the east). And as ISOL switches from BASE to INT levels, bundle combinations become more effective with little effect on cost.

**Fig 2:**
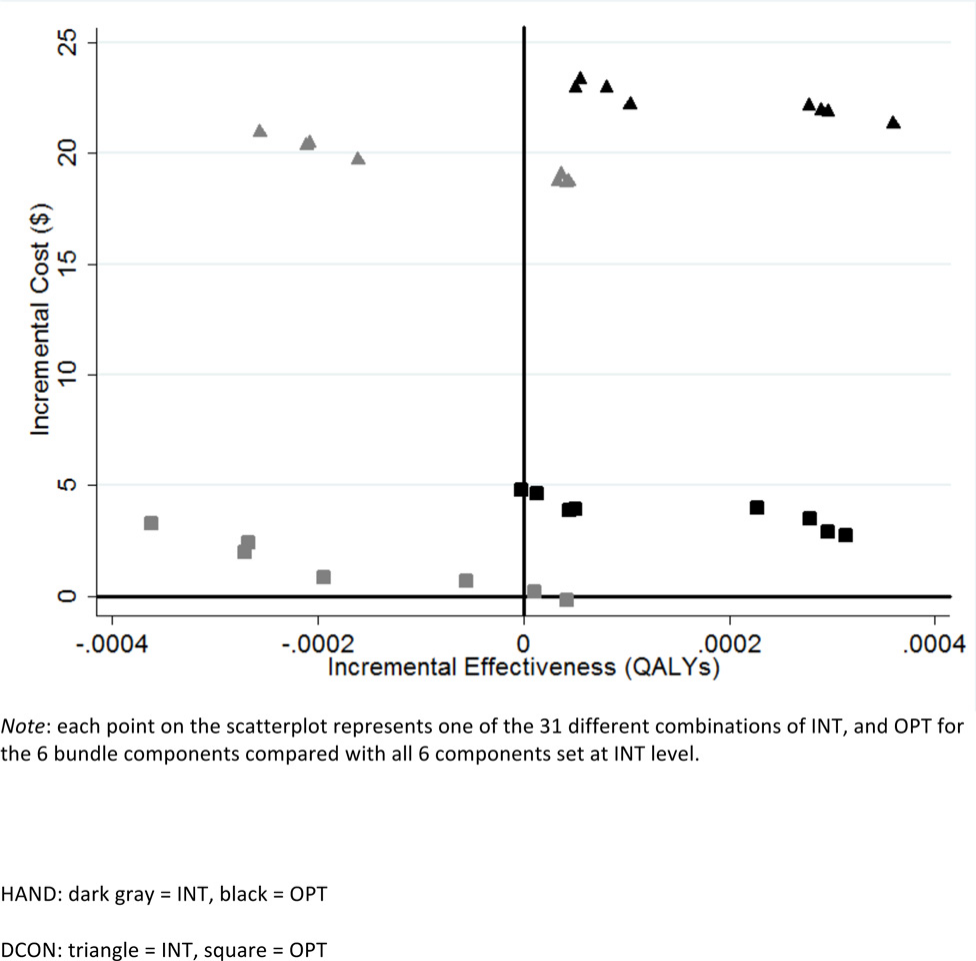
Scatterplot of incremental cost and effectiveness (*C*. *difficile* infections averted) of intervention strategies compared with INT levels of all 6 bundle components. HAND: dark gray = INT, black = OPT; DCON: triangle = INT, square = OPT.

Because ISOL only had 2 levels, 32 combinations of the remaining 5 components were possible for the comparison between OPT and INT levels. Fig 2 shows the 31 comparisons (with all components set at INT as the reference strategy) relevant for this analysis. As before, moving from INT to OPT levels for DCON decreases costs but does not change effectiveness appreciably. This same shift, in general, leads to an increase in effectiveness for HAND with a slight increase in costs.

**Fig 3:**
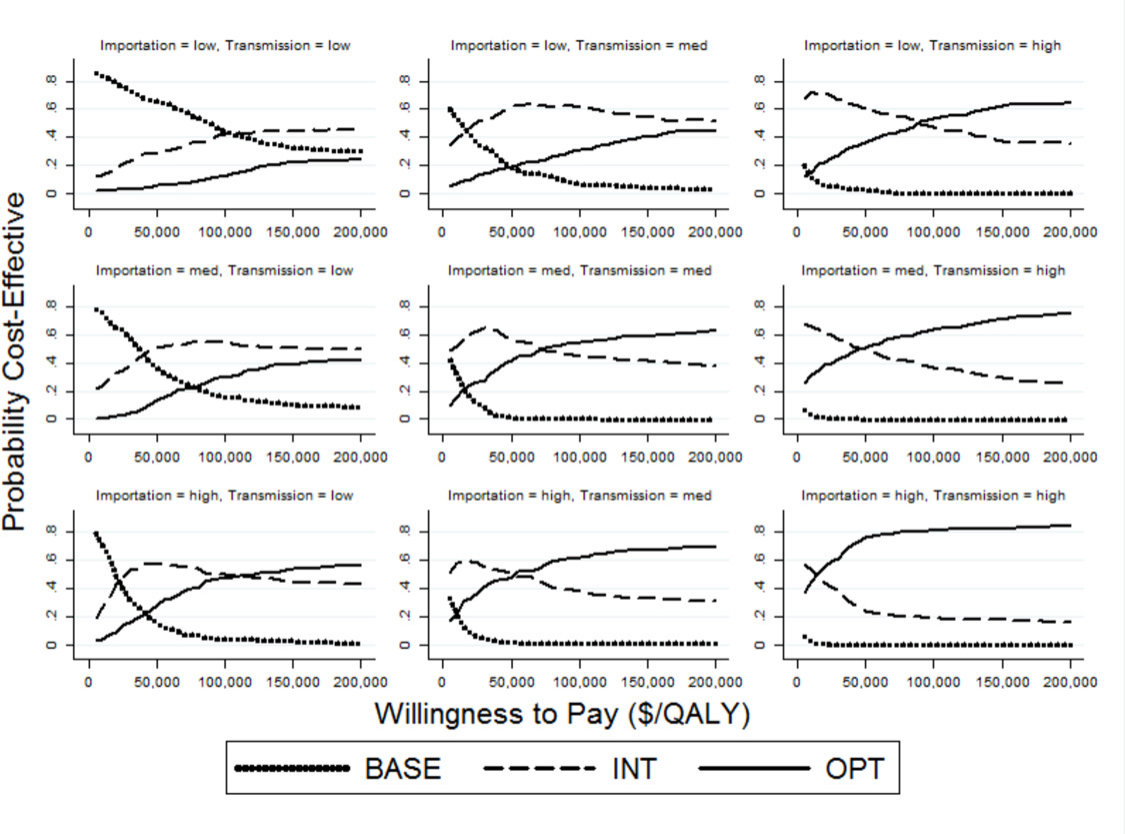
Cost-effectiveness acceptability curves depicting the probability that BASE, INT, and OPT strategies are cost-effective across different threshold values of willingness to pay from 195 2^nd^ order Monte Carlo simulations.

Finally, the results from the PSAs are shown as cost-effectiveness acceptability curves,[47,48] which depict the probability of each strategy being cost-effective at various willingness-to-pay thresholds, are shown in Fig 3 for each combination of the transmissibility and importation values. These figures show that as importation and transmission parameters increase, the probability of BASE being cost-effective decreases and the probability of OPT being cost-effective increases. For example, at a willingness-to-pay threshold of $50,000/QALY, BASE was cost-effective in 63% of the iterations when both importation and transmission were low. However, when transmission was switched to high and importation remained low or when transmission remained low and importation switched to high, INT became the strategy that was most often cost-effective (in 61% or 59% of the iterations, respectively).

## Discussion

Ours is the first dynamic cost-effectiveness analysis of strategies to prevent *C*. *difficile* transmission in the hospital. This was accomplished using an agent-based simulation model with parameter values drawn from the published literature and local and national VA data. In each comparison of bundled interventions in our analysis, increased levels of the interventions (i.e., INT compared with BASE, OPT compared with BASE, and OPT compared with INT) led to fewer CDIs. The incidence of CDI for BASE ranged from 1.8 to 19.6 per 10,000 patient-days depending on the level of importation and transmissibility of *C*. *difficile*. For medium levels of these two epidemiological parameters, the incidence of CDI was 7.5 per 10,000 patient days. To compare this to the incidence rates of healthcare-associated CDI found in the literature, we conducted a systematic literature review of multi-center studies. The pooled incidence rate across the 7 studies that we identified through this review, was 8.0 (95% CI: 6.3–11.6) per 10,000 patient days.[7,49–54]

While there were costs associated with implementing each bundle component, in the case of high transmission, we found that the reduction in costs resulting from fewer CDIs outweighed the increased costs of the INT intervention compared to BASE, yielding a net cost saving. For other levels of transmission and for all levels of importation, the INT intervention was more costly than BASE. The willingness-to-pay threshold for new healthcare interventions has historically been $50,000 per QALY in the United States.[55] However, this threshold has not been updated to take into account technology changes and inflation since its introduction several decades ago. A recent opinion piece recommended higher thresholds of $100,00 to $150,000 per QALY.[56] The increased QALYs that came from the higher costs of INT compared to BASE in our analyses led to ICERs that ranged from $616 to $80,118 per QALY, all lower than the proposed $100,000 per QALY threshold. For OPT level interventions, ICERs were greater than this threshold (ranging from $189,776 to $923,269 per QALY) for low importation or low transmission. However, OPT was cost-effective (with ICERs ranging from $73,780 to $15,628 per QALY) for transmission and importation levels of medium or higher.

Often, hospital decision makers are reluctant to engage in efforts to reduce nosocomial infections because of the anticipated costs and effort involved as well as the lack of level I evidence.[57] Our results suggest that even the expected effect from typical adherence to hospital-wide efforts to control CDI can achieve significant improvements in patient outcomes and reductions in cost without the need for extremely high adherence. In fact, our results suggest that the cost involved with setting these interventions to their optimal levels is often not worth the incremental benefit achieved. Making small improvements was sufficient to reduce infections and costs.

While this is the first cost-effectiveness analysis to compare bundled interventions for prevention of *C*. *difficile* transmission and infection, other published studies have examined the economic consequences of strategies to treat *C*. *difficile* infection or prevent transmission. Each of these studies used static analytic approaches. One such study by Lee et al. focused on a vaccine for *C*. *difficile*.[12] Because such a vaccine has not yet been introduced into clinical practice, the authors performed their analyses across a wide range of potential scenarios including vaccine efficacy and cost. They found that this vaccine would be cost-effective across many of these scenarios. Bartsch and colleagues examined the value of screening inpatient admissions for *C*. *difficile*.[15] They found that screening, which was accompanied by isolation in their model, was cost-effective at a threshold of $50,000/QALY and was economically dominant for certain colonization rates, infection probabilities, and compliance levels with contact isolation. In another study, Bartsch and colleagues examined the cost-effectiveness of fidaxomicin based on the results of strain typing compared with no fidaxomicin.[13] The authors found that, at the current cost of fidaxomicin, these treatment strategies were not cost-effective as first-line treatment either due to being dominated or having ICERs well above generally accepted willingness-to-pay thresholds. Finally, Stranges and colleagues compared the cost-effectiveness of fidaxomicin with oral vancomycin.[14] They found the fidaxomycin treatment strategy to have an ICER of $67,576/QALY.

Agent-based simulation models have been used to evaluate the cost-effectiveness of treatment strategies and interventions for other infections. For example, Schneider and colleagues constructed a stochastic agent-based model to examine the economic consequences of human immunodeficiency virus (HIV) preexposure prophylaxis.[58] They found that this strategy was cost-effective with ICERs ranging from $8399-$11,575/QALY. To our knowledge, only one other study has employed a dynamic cost-effectiveness analysis using an agent-based simulation model in the context of healthcare-associated infections. Robotham et al. used this modeling strategy to investigate the use of screening, isolation, and decolonization for methicillin resistant *Staphylococcus aureus* in intensive care units.[59]

It is important to note several limitations in this study related to a number of simplifying assumptions that we made to improve the tractability of this complicated model. First, we did not incorporate death of fulminant colitis as complications from CDI. This is most likely a conservative assumption since these complications would add to the cost and utility decrement associated with CDI, thus leading to a greater difference between the reference and intervention strategies. Second, our model assumes 100% efficacy of CDI treatment and does not consider recurrent CDI. Finally, while our model includes 6 different interventions to prevent transmission of *C*. *difficile* in a hospital setting, our model did not consider antimicrobial stewardship as a potential intervention. We are actively developing simulation models to examine this complex intervention.

In conclusion, the results from our economic analysis indicate that under most epidemiological conditions, implementing a series of bundled interventions to prevent CDI transmission at intermediate levels can reduce both inpatient costs and infections. Hand hygiene and early isolation and treatment of CDI cases were the interventions that had the greatest impact on our results. Finally, optimal levels of each intervention did not seem to provide an added benefit that outweighed the increased cost compared with intermediate levels. This work provides decision makers in hospitals with important information regarding the economic implications of infection control strategies.

## Supporting Information

S1 Appendix Detailed description of agent-based simulation model. (DOCX)
